# Improving the Quality of Health Care in Special Neonatal Care Units of India: A Before and After Intervention Study

**DOI:** 10.9745/GHSP-D-22-00085

**Published:** 2022-10-31

**Authors:** Ashok K. Deorari, Praveen Kumar, Deepak Chawla, Anu Thukral, Sonika Goel, Rajashree Bajaj, Manish Singh, Clare Gilbert, Rajan Shukla, for the Retinopathy of Prematurity-Quality Improvement India Study Group

**Affiliations:** a Department of Pediatrics, All India Institute of Medical Sciences, New Delhi, India.; b Department of Pediatrics, Postgraduate Institute of Medical Education and Research, Chandigarh, India.; c Department of Neonatology, Government Medical College and Hospital, Chandigarh, India.; d Department of Pediatrics, All India Institute of Medical Sciences, New Delhi, India.; e Postgraduate Institute of Medical Education and Research, Chandigarh, India.; f National Health Mission, Bhopal, India.; g International Centre for Eye Health, Department of Clinical Research, London School Hygiene & Tropical Medicine, London, United Kingdom.; h MCH and Health Care Quality Group, Indian Institute of Public Health, Hyderabad, India.; i Members listed at the end of the article.

## Abstract

**Background:**

We evaluated the efficacy of training health care workers (HCWs) in point-of-care quality improvement (POCQI) and a preterm newborn health care package (PHCP), followed by remote mentoring and supportive supervision in improving health care practices, neonatal survival, and morbidities in special neonatal care units (SNCUs).

**Methods:**

This pre- and postintervention quality improvement study was conducted at 3 SNCUs in Madhya Pradesh, India from February 2017 to February 2019. Clinical care teams comprising doctors and nurses from the study sites were trained in POCQI and the PHCP. The teams identified, prioritized, and analyzed problems and designed quality improvement initiatives at their respective health facilities. Change ideas were tested by the local teams using sequential plan-do-study-act cycles. Facilitators maintained contact with the teams through quarterly review meetings, fortnightly videoconferencing, on-demand phone calls, and group chat service. State SNCU coordinators made follow-up visits to supplement coaching. Study research staff independently collected data on admissions, health care practices, and outcomes of neonates.

**Findings:**

A total of 156 HCWs were trained in the POCQI methodology and PHCP. Sixteen quality improvement projects were formulated and implemented. Among 13,821 enrolled neonates (birth weight 2275±635 g; gestation: 35.8±2.8 weeks), improvement was seen in reduction of use of oxygen (36.1% vs. 48.0%; adjusted odds ratio [aOR]=0.60, 95% confidence interval [CI]=0.55, 0.66), antibiotics (29.4% vs. 39.0%; aOR=0.76, 95% CI=0.68, 0.85), and dairy milk (33.8% vs. 49.4%; aOR=0.34, 95% CI=0.31 to 0.38). Enteral feeds were started within 24 hours of admission in a larger number of neonates, resulting in fewer days to reach full feeds. There was no effect on survival at discharge from the hospital (aOR=0.93; 95% CI=0.80, 1.09).

**Conclusion:**

A collaborative cross-learning quality improvement approach with remote mentoring, coaching, and supportive supervision was successful in improving the quality of care at SNCUs.

## Abstract (precis)

In low- and middle-income countries, facility-based neonatal care practices can be improved using a collaborative cross-learning quality improvement approach incorporating remote mentoring, coaching, and supportive supervision.

## Teaser

**Key Findings:**

- Combining a training package to improve the knowledge and skills of health care workers in managing sick and preterm neonates with point-of-care quality improvement skills successfully reduced the unindicated use of oxygen, antibiotics, and phototherapy while increasing the use of enteral feeds.
- Despite a significant reduction in the use of 2 lifesaving medical interventions—oxygen and antibiotics—no increase in mortality or short-term adverse outcome was observed, indicating an overuse of these interventions in the preintervention period.

**Key Implication:**

- A strategy of combining professional training activities and coaching in continuous quality improvement methods can successfully improve the health care of sick and preterm neonates. With the rapid expansion of access to facility-based neonatal care, it is important to monitor for both overuse and underuse of evidence-based interventions.

## INTRODUCTION

Improved access to facility-based neonatal care (FBNC) has enabled sick and preterm neonates to receive level-2 clinical services in district hospitals in India. Special newborn care units (SNCUs) at district hospitals provide FBNC to about 0.9 million neonates every year, on approximately 16,000 beds across India.^1^ Common reasons for admission include birth asphyxia, encephalopathy, low birth weight or preterm birth, sepsis, and hyperbilirubinemia.^2^ Medical care available at the typical SNCU includes resuscitation at birth, thermal care, oxygen therapy, phototherapy, gavage feeding, kangaroo mother care, intravenous fluid administration, antibiotic therapy, and detection and referral for congenital abnormalities. An increasing number of SNCUs provide continuous positive airway pressure therapy to neonates with respiratory distress. SNCUs also offer postdischarge follow-up services to high-risk neonates and refer infants needing intervention to District Early Intervention Centers or medical colleges. Along with an increase in institutional births, FBNC has contributed to a substantial reduction in the neonatal mortality rate during the last decade.^3^ However, unexplained variations in survival to discharge, the incidence of morbidities, and utilization of interventions among the SNCUs indicate the scope for quality improvement (QI).^4^^,^^5^

Although the SNCUs have adequate infrastructure and supplies, concerns have been raised about suboptimal knowledge and poor skills of the health care workers (HCWs) that staff them.^6^ Evidence on the effectiveness of various strategies to improve neonatal care practices of HCWs in low- and middle-income countries is limited, heterogeneous, and of low quality.^7^ While suboptimal knowledge and poor skills indicate a need for augmentation, strategies like distribution of printed educational material, training workshops, technology-based interventions, and supervision alone have no or minimal effect in improving health care practices.^7^ Group problem-solving strategies have shown moderate to large benefits, but the HCWs in India do not receive preservice training in QI methods.^8^ Earlier reports from India about QI in neonatal care have focused on isolated care processes and originate from tertiary care medical colleges.^9^^–^^13^ There is a need to evaluate whether combining knowledge and skills augmentation activities with problem-solving skills can improve the ability of HCWs to adapt and adopt evidence-based practices and improve health outcomes in SNCUs. Knowledge and skill augmentation can improve health care by increasing the know-how about a health condition (e.g., what are appropriate oxygen saturation targets in preterm neonates and what are methods of oxygen administration). Problem-solving skills can improve health care by enabling HCWs to identify, analyze, and address local health system constraints which preclude the application of updated knowledge and skills (e.g., monitoring oxygen saturation in preterm neonates using the available human resources and pulse oximeters).

Can combining knowledge and skills augmentation activities with problem-solving skills improve HCWs’ ability to adapt and adopt evidence-based practices and improve health outcomes in SNCUs?

**FIGURE 1:**
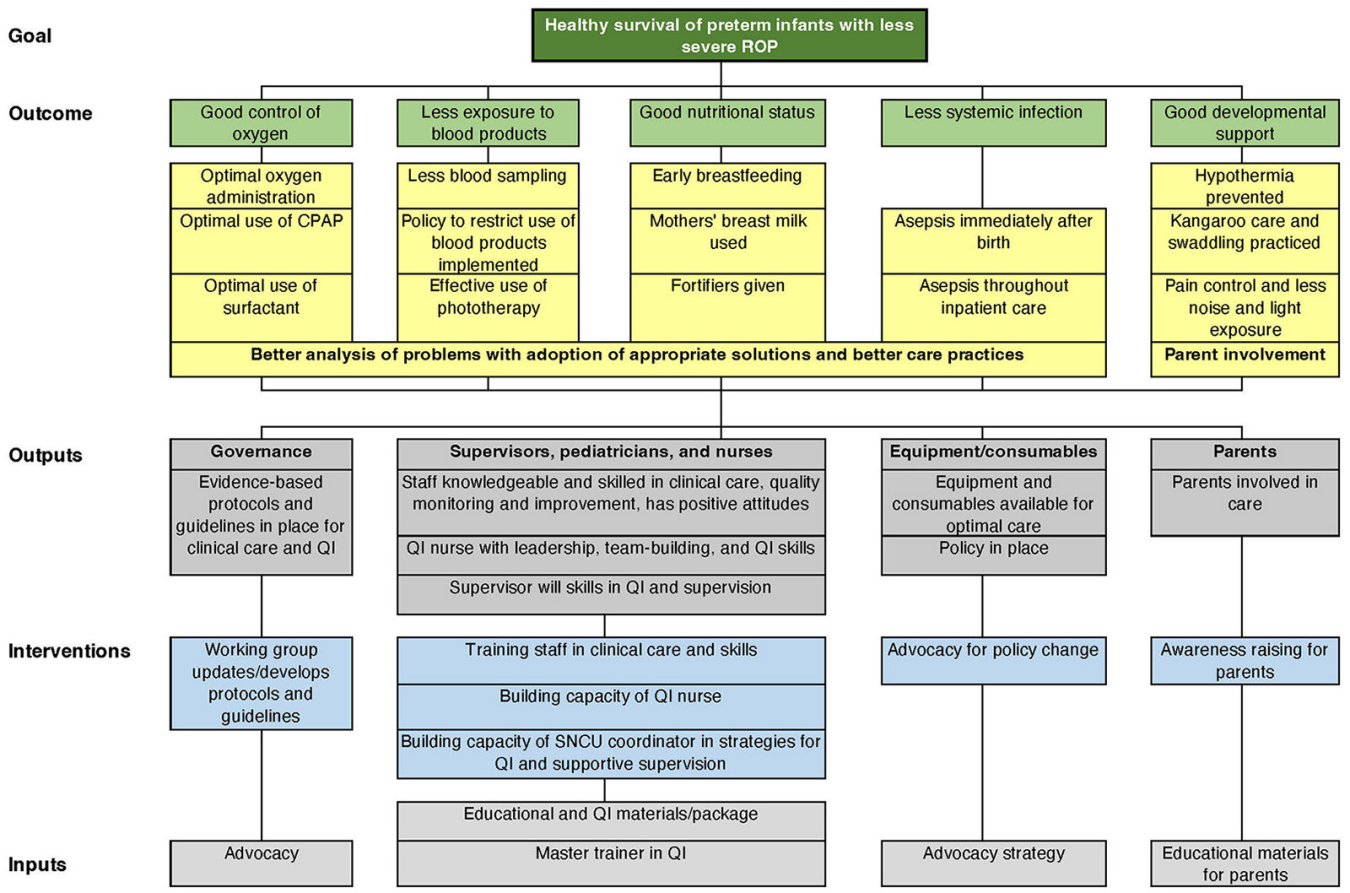
Theory of Change for a Special Neonatal Care Unit Point-of-Care Quality Improvement Intervention, Madhya Pradesh, India Abbreviations: CPAP, continuous positive airway pressure therapy; QI, quality improvement; ROP, retinopathy of prematurity; SNCU, special newborn care unit.

Our study evaluated whether the implementation of an intervention package to improve the knowledge and skills of the HCWs in providing clinical care as well as undertaking QI activities would improve health care practices, neonatal survival, and morbidities in the SNCUs. To test this hypothesis, we developed a theory of change that formed the basis of the package of interventions (Figure 1).^14^ The theory of change focused on reducing modifiable risk factors for retinopathy of prematurity (ROP), a common cause of acquired blindness in children, which is an excellent indicator of the quality of care provided to preterm neonates in a health care facility. Risk factors for ROP include unmonitored and unnecessary oxygen administration, sepsis, poor nutrition, and exposure to blood products.^15^^–^^17^ Therefore, interventions aimed at reducing ROP are likely to affect many aspects of neonatal health care resulting in improved health outcomes. This article focuses on the main results of the intervention.

## METHODS

**FIGURE 2:**
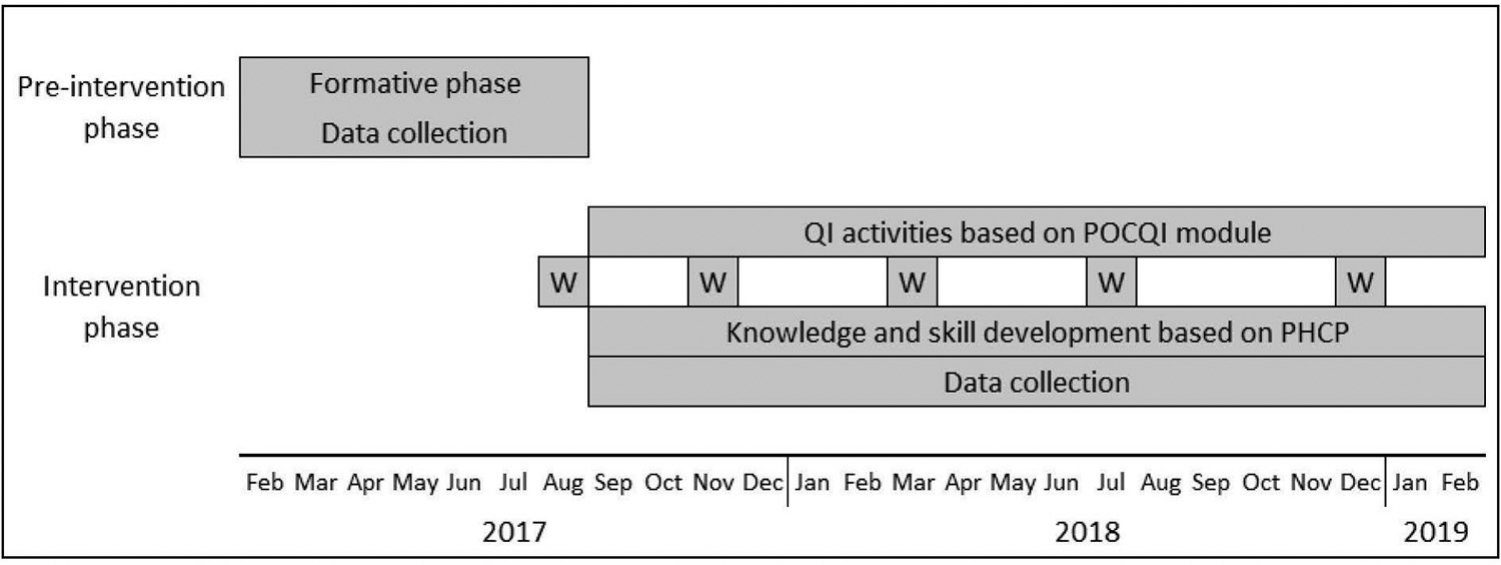
Phases and Activities of a Special Neonatal Care Unit Point-of-Care Quality Improvement Intervention, Madhya Pradesh, India Abbreviations: PHCP, preterm newborn health care package; POCQI, point-of-care quality improvement; W, workshop.

This before-and-after study was conducted from February 2017 to February 2019 in the SNCUs of 3 district hospitals and a medical college in the state of Madhya Pradesh, India (Figure 2). All liveborn neonates admitted in the SNCUs for at least 24 hours, who died within 24 hours of admission or who were referred to a tertiary care hospital within 24 hours of admission were eligible to participate in the study. Madhya Pradesh was 1 of the 4 states selected for the project supported by the Queen Elizabeth Diamond Jubilee Trust (2013–2019) following a situational analysis. Madhya Pradesh, the second largest state in India, ranks poorly in health indicators and is 1 of the high-focus states for interventions by the federal government’s health programs. Madhya Pradesh was one of the first states to establish SNCUs in district hospitals with the support of UNICEF. SNCUs at district hospitals have similar infrastructure and human resources. A district SNCU has 12–20 beds and provides 24-hour care with 1 doctor, 2 nurses, and 1 support staff in each shift.^18^ Of the total 3–4 doctors stationed at SNCU, at least 1 is a pediatrician and is the overall in-charge. SNCUs at the medical colleges have more human resources, including faculty and postgraduate students, and are equipped to deliver a higher level of care including assisted ventilation. A local medical college at Indore was chosen to mentor the QI activities at the included SNCUs. However, due to the high workload and a lack of mentoring capacity in the QI field, this role had to be assumed by the coordinating sites of the study. Midway during the study (May 2018), the medical college at Bhopal was instead engaged as the local mentor institution.

### Ethical Approval

The study was coordinated by the All India Institute of Medical Sciences, New Delhi, the Postgraduate Institute of Medical Education and Research, Chandigarh, and the Government Medical College Hospital, Chandigarh. Institutional ethics committees at the 3 coordinating sites and the Indian Institute of Public Health, Hyderabad approved the study protocol. National Health Mission, Madhya Pradesh also approved the study protocol. Written informed consent was obtained from parents of the enrolled neonates, HCWs at the study sites, state SNCU coordinators, and state child health consultants.

### Study Work Plan

The study intervention was guided by a theory of change and the work plan was divided into preintervention and intervention phases.

#### Preintervention Phase

The preintervention phase had 2 components—formative assessment and baseline neonatal data collection. The formative component started with the development of a theory of change (Figure 1) based on targeting the following risk factors for the development of ROP in low- and middle-income countries: unmonitored and unnecessary oxygen administration, systemic infection, poor nutrition, lack of developmentally supportive care, and exposure to blood products.^19^^–^^22^ Starting with these risk factors, we identified the primary drivers and downstream health care activities that can lead to improvement in survival without severe ROP (Box).

BOX **Strategies to Improve ROP-Free Survival Outcomes**

Optimize oxygen use:

- Improve oxygen administration methods
- Monitor and promote the use of continuous positive airway pressure and surfactant

Reduce exposure to blood products:

- Minimize amount of blood sampling
- Restrict use of blood products
- Effective use of phototherapy

Improve nutritional status:

- Early initiation and rapid advancement of enteral feeding with breast milk

Reduce sepsis:

- Improve aseptic techniques at birth and during hospital admission

Improve developmental care:

- Hypothermia prevention
- Kangaroo mother care
- Pain control
- Reduce noise and light exposure
- Increase parental involvement in care

In parallel, we assessed the baseline levels of knowledge, skills, attitudes, and practices of SNCU health care personnel, state SNCU coordinators, and child health consultants. This preassessment focused on the clinical care pathways including the drivers of improvement identified in the theory of change. We also assessed the problem-solving and QI skills of the HCWs. Results of this formative part of the study are being reported separately. Based on this formative phase, we designed, pilot-tested, and finalized a collection of interventions with 2 components: (1) improvement in knowledge and skills of the HCWs in neonatal care and (2) improvement in the ability of staff to practice the knowledge and skills gained by conducting QI activities at their respective SNCUs. The content for training HCWs on best practices in preterm care was called the preterm newborn health care package (PHCP) and included 10 modules composed of brief webinars, videos, case studies, posters, job aids, and key messages. The content of each module was divided into specific learning objectives, with a self-assessment in the form of multiple-choice questions following each objective. The evidence-based content and materials in the PHCP (www.pretermcare-eliminatingrop.com) were developed and reviewed by 35 neonatologists and nurses from across the country. The Point of Care Quality Improvement (POCQI) tool, developed in collaboration with the World Health Organization South-East Asia Regional Office (WHO-SEARO), guided the training for conducting QI projects.^23^ These tools (PHCP and POCQI) included reading material, practical exercises, and knowledge and skill assessment instructions for both the learners and facilitators. The use of these tools ensured homogeneity and consistency of the mentoring activities during the study. During the preintervention phase, baseline neonatal health data were collected from the study SNCUs over 7 months (February 2017 to August 2017) parallel to the formative phase of the study.

The evidence-based content and materials in the PHCP were developed and reviewed by 35 neonatologists and nurses from across the country.

#### Intervention Phase

**In-person training workshops:** We rolled out the intervention with a series of on-site small-group training and QI project development workshops. The HCWs participated in the training workshops in small batches of 1–2 doctors and 2–3 nurses from each SNCU. Each workshop was held over 2.5 days and was divided into 2 parts. The first part consisted of knowledge and skill augmentation in 2 of the available modules. These modules were identified and developed for training based on the theory of change and formative research. The available modules included thermoregulation, kangaroo mother care, optimal oxygen administration, developmentally supportive care and pain management, less exposure to blood products and prevention of exchange transfusion, delivery room management and stabilization of a preterm infant, optimal use of continuous positive airway pressure, less systemic infections, good nutrition, and follow-up care pertaining to ROP. The modules were designed as self-learning activities supplemented by skill training. Each workshop only covered 2 modules in order to fit within 2.5 days (all the training materials, including the remaining 8 modules, were also made available on the website for ongoing training). Activities for knowledge and skill augmentation included small group discussions, skill practice, and simulated clinical scenarios. The second part consisted of clinical data presentation from each SNCU, choosing target areas of improvement, forming QI teams, analyzing the root causes of the problem, and outlining the QI project activities. Each SNCU team not only worked on the development of its own QI project but also reviewed and gave feedback on the QI projects of other SNCUs. During the study, selected HCWs from each SNCU underwent a total of 5 in-person sessions that covered all the modules developed under the PHCP.

**Ongoing support:** In addition to the training in the remaining modules during successive in-person workshops, all the training materials were available online for ongoing training and review. Skill training corners were also established in each SNCU, and a skill lab was established in the mentoring medical college.

**QI projects and mentoring:** The theory of change guided the selection of QI projects. Doctors and nurses working in each SNCU selected specific problems to be addressed by a QI project and decided the goal and outcome measures for each project based on the 4 criteria outlined in the POCQI guide—importance to patient outcomes, affordability in terms of time and resources, ease of measurement of outcome, and whether the identified problem was under the control of team members. Following the workshop, the SNCU teams returned to their units and started working on their respective QI projects. We held web meetings every two weeks to review progress and provide guidance for any bottlenecks encountered. Team members presented their completed projects in subsequent on-site workshops after which they developed and conducted new QI projects. The intervention period continued from September 2017 to February 2019 and over this period each SNCU conducted 3–5 QI projects (Supplement, Table S1).

### Study Outcomes

A study research nurse at each of the 3 SNCUs collected the health outcome data of the admitted neonates. The primary outcome of the study was the proportion of neonates who survived without developing severe ROP. Secondary outcomes included death before discharge, adverse outcomes (death, referral to a higher center, or left against medical advice), hypothermia at admission, neonatal hyperbilirubinemia needing phototherapy or exchange transfusion, and duration of hospital stay. Various care processes reported include enteral feeding practices, use of oxygen and antibiotics, and screening for ROP.

The primary outcome of the study was the proportion of neonates who survived without developing severe ROP.

### Statistical Analysis

We defined the composite primary outcome as neonates discharged alive from SNCU and not found to have severe ROP on screening examination before discharge or during follow-up. We defined severe ROP as ROP needing laser ablation or surgery to prevent loss of vision. Although estimates of mortality among SNCU admissions are available, no such information is available about the incidence of severe ROP, the second component of the composite primary outcome. Therefore, the sample size was calculated for the incidence of mortality among SNCU admissions. With an estimated enrollment of 3,000 neonates in the preintervention period and 10,000 neonates during the intervention period, the study had 90% power to detect an absolute reduction of 2% in mortality (from 10% to 8%) with a 2-sided alpha of 0.05.

Study nurses prospectively collected data from the bedside clinical records and entered it into a custom-built Android application. We then analyzed the data using Stata 16.1. Categorical data are presented as numbers and percentages. Normally distributed numerical data are presented as mean and standard deviation, and skewed numerical data are presented as median with interquartile range. We used a mixed-effects regression model to estimate the effect of the intervention, with SNCU site as random effects and other covariates (gestation at birth, gender, presence of congenital malformation, antenatal care, outborn status, and need for positive pressure ventilation at birth) as fixed effects. The covariates included in the model were based on biological plausibility and significant differences between the preintervention and intervention periods. We conducted a post-hoc secondary analysis to account for the possible effect of a secular trend in the preintervention and intervention periods. For this, we aggregated the data for each month and then analyzed the data using the interrupted time-series regression method which separates the effect of intervention from the preintervention trend. A *P* value of less than .05 was considered statistically significant and no adjustment was made for multiple comparisons.

## RESULTS

### Study Flow and Input

Over the study period, a total of 5 in-person sessions were held and 156 HCWs were trained. A total of 16 QI projects were completed at the 3 study SNCUs of the district hospitals and 1 SNCU of a medical college (Supplement, Table S1 and Table S3). All the doctors and >90% of nurses in the study SNCUs received training over the period of intervention. All the learning modules were covered over the 5 in-person sessions and the continuous online training and mentoring. The flow of online modules on the web portal ensured that the participants were able to proceed to the subsequent module only after successfully completing the knowledge assessment of the current module. The skill check activities were directly observed. Due to the midway change in the mentoring medical college, preintervention data were not available from the SNCU in Bhopal and so data from this site were not included in our primary analysis. Throughout the study, the 3 SNCUs were mentored by investigators from outside the state of Madhya Pradesh.

### Baseline Characteristics

A total of 13,821 neonates were enrolled in the study. The mean birth weight of the study cohort was 2275±635 g and the mean gestation was 35.8 ± 2.8 weeks. A majority of neonates were outborn (8,450, 61.2%). Antenatal care was almost universal, with 87% of mothers having had at least 3 antenatal check-ups and 11.2% having had cesarean delivery. A high proportion (43.6%) of neonates were preterm (<37 weeks of gestation). Over the study period, the profile of enrolled neonates remained similar (Table 1), although the proportion of preterm births increased.

**TABLE 1.** Baseline Variables of a Special Neonatal Care Unit Point-of-Care Quality Improvement Intervention, Madhya Pradesh, India

	**Preintervention Period**	**Intervention Period**
Admission/center, No./Total (%)		
Dhar	875/3352 (26.1)	3089/10469 (29.5)
Ujjain	1294/3352 (38.6)	3770/10469 (36.0)
Sehore	1183/3352 (35.3)	3610/10469 (34.5)
Birth weight, mean±SD, g	2274±628	2276±638
Birth weight category, No./Total (%)		
≥2500 g	1366/3346 (40.8)	4208/10462 (40.2)
1500–2499 g	1585/3346 (47.4)	5043/10462 (48.2)
1000–1499 g	325/3346 (9.7)	1025/10462 (9.8)
<1000 g	70/3346 (2.1)	186/10462 (1.8)
Gestation, mean±SD, weeks	35.9±2.9	35.7±2.8
Preterm birth, No./Total (%)	1401/3343 (41.9)	4624/10460 (44.2)
Gestation age category, No./Total (%)		
>37 weeks	1942/3343 (58.1)	5836/10460 (55.8)
34–36 weeks	849/3343 (25.4)	2851/10460 (27.3)
<34 weeks	552/3343 (16.5)	1773/10460 (17.0)
Male gender, No./Total (%)	1478/2843 (52.0)	5920/10412 (56.9)
Outborn, No./Total (%)	2086/3348 (62.3)	6364/10457 (60.9)
Antenatal care, No./Total (%)	3266/3349 (97.5)	10370/10445 (99.3)
At least three antenatal visits, No./Total (%)	2838/3144 (90.3)	8787/10271 (85.6)
Maternal hypertension, No./Total (%)	25/3250 (0.8)	31/10408 (0.3)
Oligohydramnios, No./Total (%)	21/3103 (0.7)	21/10097 (0.2)
Antenatal steroids if gestation <35 weeks, No./Total (%)	157/811 (19.4)	359/2177 (16.5)
Cesarean delivery, No./Total (%)	358/3352 (10.7)	1203/10469 (11.5)
Multiple pregnancy, No./Total (%)	88/3352 (2.6)	264/10469 (2.5)
Positive pressure ventilation at birth, No./Total (%)	123/3352 (3.7)	463/10469 (4.4)
Intubation at birth, No./Total (%)	3/3352 (0.1)	19/10469 (0.2)

Abbreviations: SD, standard deviation.

### Study Outcomes

A total of 343 (10.2%) neonates died during the preintervention period and 1,106 (10.6%) during the intervention period (adjusted odds ratio [aOR]=0.93; 95% confidence interval [CI]=0.80, 1.09). As gravely sick neonates may be referred for tertiary care or taken home by parents for compassionate care, we also calculated the rate of adverse outcomes including death, referral to a higher center, or left the hospital against medical advice. No difference was observed in the incidence of adverse outcomes (aOR=1.01; 95% CI=0.90, 1.15).

Several health care practices improved during the intervention period (Table 2). These included an increase in the proportion of neonates in whom temperature was measured at admission and enteral feeds were started within 24 hours of admission. The use of many interventions declined, indicating overuse in the preintervention period, including a significant decrease in the use of oxygen, dairy milk, phototherapy, and antibiotics. A significantly lesser proportion (36.1% vs. 48%) of neonates received oxygen therapy at admission in the intervention period (Table 2). This reduction was observed in neonates born at term (>36 weeks, 33.7% vs. 43.9%), late preterm (34–36 weeks, 37% vs. 51.1%, *P*<.01), and very preterm (<34 weeks, 42.6% vs. 57.8%, *P*<.01) gestation. The duration of oxygen therapy also decreased (Table 2). A significant reduction was also noted in the use of systemic antibiotics (29.4% vs. 39%). In the domain of nutrition, a significantly higher proportion of neonates were started on enteral feed within 24 of birth or admission, use of dairy milk was reduced, and neonates reached full enteral feeding at an earlier age in the intervention period (Table 2). The proportion of neonates who received phototherapy decreased significantly without an increase in the proportion of neonates who developed serum bilirubin of more than 20 mg/dL. The proportion of eligible neonates who underwent ROP screening at the SNCU was reduced. The most common reason for not being screened for ROP included the unavailability of an ophthalmologist and parents not being aware of the need for screening after discharge from the SNCU (Supplement, Table S2).

**TABLE 2.** Outcome Variables of a Special Neonatal Care Unit Point-of-Care Quality Improvement Intervention, Madhya Pradesh, India

	**Preintervention Period, No./Total (%)**	**Intervention Period, No./Total (%)**	**Adjusted**^a^ **Odds Ratio or Mean Difference (95% CI)**
Death before discharge	343/3352 (10.2)	1106/10469 (10.6)	0.93 (0.80, 1.09)
Adverse outcome^b^	519/3352 (15.5)	1761/10469 (16.8)	1.01 (0.90, 1.15)
Oxygen started at admission	1608/3352 (48.0)	3778/10469 (36.1)	0.60 (0.55, 0.66)
CPAP started at admission	69/3352 (2.1)	186/10469 (1.8)	0.80 (0.58, 1.10)
Temperature at admission	2958/3352 (88.2)	9648/10469 (92.2)	1.54 (1.33, 1.79)
Enteral feeds started within 24 hours of admission	1254/3217 (39.0)	4250/10360 (41.0)	1.17 (1.07, 1.29)
Breast milk as first feed	922/3352 (27.5)	2961/10469 (28.3)	1.10 (0.99, 1.22)
Use of dairy milk for feeding	1655/3352 (49.4)	3535/10469 (33.8)	0.34 (0.31, 0.38)^c^
Use of phototherapy	684/3115 (22.0)	1838/10095 (18.2)	0.82 (0.74, 0.92)
Peak serum bilirubin >20 mg/dL	14/3352 (1.9)	36/10469 (1.8)	1.02 (0.50, 2.10)
Use of any systemic antibiotic	845/2166 (39.0)	2982/10315 (29.4)	0.76 (0.68, 0.85)
Reach full enteral feeds, No. (95% CI), days	8.0 (5.0, 12.0)	6.0 (3.0, 11.0)	−2.2 (−4.00, −0.40)
Oxygen therapy, No. (95% CI), days	2.0 (1.0, 2.0)	1.0 (1.0, 2.0)	−0.3 (−0.50, −0.20)
ROP screening^d^	320/2052 (15.6)	483/6410 (7.5)	0.44 (0.37, 0.52)

Abbreviations: CI, confidence interval; CPAP, continuous positive airway pressure; ROP, retinopathy of prematurity.

^a^Adjusted for center, gestation at birth, gender, presence of congenital malformation, antenatal care, outborn status, and need of positive pressure ventilation at birth.

^b^Death, left against medical advice, or referred.

^c^Simple logistic regression as mixed effects model did not fit.

^d^Among neonates with birth gestation less than 37 weeks or weight less than 2000 g.

The use of many interventions, including oxygen and antibiotics, declined, indicating overuse in the preintervention period.

The interrupted time-series analysis adjusting for the preintervention trend showed a significant reduction of 2.2% (95% CI=−3.4, −1.1) per intervention month in the proportion of neonates who were started on oxygen at admission and a significant reduction of 5.1% (95% CI=−9.6, −0.6) per intervention month in the proportion of neonates who received antibiotics (Table 3; Supplement, Figures S1 and S3). The trend-adjusted analysis showed no significant change in other health care practices, death, or adverse outcomes (Supplement, Figure S4).

**TABLE 3.** Interrupted Time-Series Trend Analysis of the Effect of a Special Neonatal Care Unit Point-of-Care Quality Improvement Intervention, Madhya Pradesh, India

	**Preintervention Period Trend, %/Month (95% CI)**	**Effect During the First Month of Intervention, % (95% CI)**	**Trend During Intervention Period (Relative to Preintervention Period), %/Month (95% CI)**
Death	0.36 (−0.25, 0.98)	−1.2 (−3.9, 1.5)	−0.39 (−1.00, 0.30)
Adverse outcome	0.20 (−0.01, 0.49)	−0.25 (−2.58, 2.07)	−0.10 (−0.46, 0.27)
Oxygen started at admission	1.39 (0.46, 2.32)	−9.54 (−15.6, −3.4)	−2.24 (−3.35, −1.13)
Temperature at admission	1.78 (−0.37, 3.94)	−4.1 (−10.3, 2.0)	−1.6 (−3.80, 0.56)
Enteral feeds started within 24 hours of admission	−1.5 (−3.3, 0.35)	13.6 (2.0, 25.2)	0.7 (−1.40, 2.80)
Breast milk as first feed	0.5 (−1.7, 2.7)	−0.2 (−9.8, 9.4)	−0.5 (−2.80, 1.80)
Use of dairy milk for feeding	−1.4 (−4.5, 1.8)	−6.7 (−17.5, 4.2)	0.7 (−2.50, 4.00)
Use of phototherapy	0.4 (−2.6, 3.4)	−4.2 (−14.9, 6.6)	−0.4 (−3.40, 2.60)
Peak serum bilirubin >20 mg/dL	−0.2 (−0.5, 0.1)	−0.1 (−2.0, 1.8)	0.3 (−0.01, 0.60)
Use of any systemic antibiotic	4.9 (0.4, 9.4)	−23.2 (−46.2, −0.2)	−5.1 (−9.60, −0.60)
ROP screening	−0.2 (−0.7, 0.3)	−3.9 (−5.5, −2.4)	0.2 (−0.40, 0.70)

Abbreviations: CI, confidence interval; ROP, retinopathy of prematurity.

## DISCUSSION

We evaluated the efficacy of a novel package of interventions in improving the health care practices and outcomes in district level-2 neonatal care units. We observed significant improvement in the health care provided to sick or preterm neonates at admission and during the hospital stay. However, we did not observe any improvement in neonatal survival. Our study intervention was based on the assumption that unexplained variation in the care provided to neonates admitted to the SNCUs is related to the quality of care. During the baseline period of observations, we found that many health care interventions were often being used without clear indications. These included administration of oxygen, intravenous fluids, antibiotics, and phototherapy. The root causes of injudicious use of these interventions were lack of standard operating procedures and fear of adverse outcomes in absence of use (Supplement, Table S3). An additional challenge was the state government practice of measuring performance evaluation of the SNCUs by the number of admissions and use of various medical interventions. In our formative evaluation, this was associated with unnecessary admissions, delayed discharge, and overuse of medical interventions. During the intervention period, the SNCU teams identified and ran QI cycles to address these issues. Combined with the PHCP to improve the knowledge and skills of HCWs in managing sick and preterm neonates, the QI cycles successfully reduced the use of oxygen, antibiotics, and phototherapy while enteral feeds were initiated earlier resulting in a shorter time to reach full enteral feeds. Significant reduction in the use of 2 lifesaving but double-edged interventions like oxygen and antibiotics without a concomitant increase in the incidence of death or referral to a higher center supports our preintervention assessment of overuse. Alternatively, the profile of admitted infants may have changed over time, resulting in a lower use of oxygen and antibiotics. However, this alternate explanation is refuted by the similarity of baseline variables and significant effect size obtained even after adjusting for biologically plausible prognostic factors. Rather, the case mix in the intervention period had a higher proportion of preterm and male neonates and lower use of antenatal steroids, all of which are risk factors for increased sickness and mortality.^24^ The study did not demonstrate any reduction in mortality despite improvement in the care practices. Staffing levels or nurse-to-patient ratio did not change during the project. Lack of effect on mortality can be explained by 3 factors. First, the QI projects focused on the care provided in the SNCU. Intrapartum care and resuscitation practices have a large influence on early neonatal survival. The QI projects in this study did not target obstetric health care providers or intrapartum and resuscitative care.^25^ Second, about 60% of neonates were outborn, and the care received before admission to the hospital was not in the purview of our QI interventions. Third, neonatal mortality is influenced by many proximal and distal socioeconomic, biological, and health system–related factors. Previous efforts to reduce neonatal mortality by QI at individual units have met with mixed success.^5^^,^^7^ In a similar study, Gilbert et al. reported improvement in knowledge and practices of neonatal nurses but no change in survival, severe ROP, or other outcomes.^26^ The fragile health systems in low- and middle-income countries may need novel interventions or additional QI efforts at the macro and meso levels to improve the survival of preterm neonates.^24^

The study did not demonstrate any reduction in mortality despite improvement in the care practices.

Irrational use of oxygen, intravenous fluids, and antibiotics can not only cause harm to the neonates by increasing the risk of ROP and hospital-acquired sepsis but can also lead to increased length of stay, overcrowding, and cost of care, as well as unnecessary work for health care providers.^27^^,^^28^ We planned to evaluate the effect of the interventions on the incidence of severe ROP but this was not possible as screening services were not available at 1 study site (Sehore) for most of the study period, and at 2 other sites for part of it. As a result, a large proportion of neonates were not screened for ROP. A lack of trained ophthalmologists at peripheral hospitals has been reported previously and there is a need to evaluate alternate models of ROP screening at district hospitals such as retinal imaging with remote analysis using telemedicine and training newborn care providers in indirect ophthalmoscopy.^29^^,^^30^

The type of training materials and their mode of delivery was decided in consultation with HCWs. The local QI teams identified and prioritized which quality issues to address. The QI teams were also responsible for conducting the root-cause analysis and collecting the data. In all the SNCUs, nurses were the majority members of the QI teams and led most of the projects, with the support of the doctors. We believe that this enhanced the ownership of the intervention among HCWs at the district SNCUs and therefore resulted in significant improvement in health care practices. As all the district SNCUs in India have been designed, equipped, and staffed similarly, and follow similar FBNC packages and government guidelines, the intervention package has good external applicability.^18^

We believe that HCW ownership of the intervention was enhanced by involvement of nurses as majority members of the QI teams.

The current study highlights many of the implementation challenges that constrain the application of evidence-based health care practices. The method used in the study was a point-of-care quality improvement approach to bring changes in the health care processes, which were planned and tested by teams of HCWs. While this approach can identify and address the gaps in the processes of care (e.g., lack of standard treatment protocols), it is not able to address the gaps in the structure of the health system (e.g., availability of human and nonhuman resources or equipment, shortage of space, etc.).^31^ This fragility of the health system is one of the major challenges faced in the implementation of evidence-based interventions in resource-limited settings.^32^ Even short-term imbalances between demand and supply (e.g., due to transfer-out of HCWs, interrupted supply of lifesaving medicines, and infectious disease outbreaks) can decrease or reverse the improvements in health outcomes.^33^ In the current study suboptimal functional status of both referring and referral units resulted in overcrowding at the study SNCUs. Newborn stabilization units (level 1) were nonexistent or lacked resources and therefore referred even neonates with transient illnesses to SNCUs (level 2). On the other hand, designated neonatal intensive care units (level 3) were either located at a long distance or were not better equipped than SNCUs. Thus, parents and health care staff were reluctant in referring neonates to level 3 units. Another implementation challenge is ensuring sustainability of the intervention especially when the intervention is designed and tested by stakeholders external to the health system. To safeguard against this, the state National Health Mission functioned as the co-owner of the initiative right from the start. The state National Health Mission served as the host, part-funder, and organizer of all the POCQI and PHCP learning workshop sessions. The state medical college was designated as a local mentor to continue the training and refresher sessions for the SNCU HCWs. Informal feedback and interactions with state health officials, district SNCU doctors, and nurses indicate that many of the good practices have been adopted by the state and have been implemented in other district SNCUs of the state as well. The parameters of performance evaluation of SNCUs by the state government have also changed and include a more comprehensive assessment rather than only considering the number of admissions and use of different therapies.

### Limitations

The study has certain limitations. The first major limitation is that due to the lack of consistent availability of ophthalmologists at the district hospitals, only a subset of eligible preterm neonates could be screened for the development of ROP. Therefore, the study was not able to report any change in the incidence of severe ROP, a component of the composite primary outcome of the study. Second, being an uncontrolled study, the role of secular trends and external influences cannot be ruled out. We used an interrupted time-series regression analysis to control for the preintervention trend. Although this reduced the number of practices that were found to have improved significantly, this can be explained by reduced power as aggregated data were used in the analysis instead of individual patient data. Third, working on 1 QI project could have led to improvement in another clinical domain and the confounding effect of QI projects on each other cannot be removed. However, the QI teams at each SNCU worked on only 1 QI project at a time and only after completing the project took up the next QI project using the new baseline level of performance at that time. Fourth, the QI activities in the study focused only on the health care processes within the SNCUs. Obstetric practices, resuscitation, and, if outborn, care during transport, have a major influence on neonatal mortality and morbidity. This limitation may be a major reason for the study intervention not resulting in improved neonatal survival. Fifth, the study did not formally evaluate the sustainability of the effects of the intervention package. It is possible that the withdrawal of the external collaborators could result in reverting to previous health care practices. Lastly, we have not evaluated the cost-effectiveness of the intervention.

Focusing only on health care processes within the SNCUs may be a major reason that the study intervention did not result in improved neonatal survival.

## CONCLUSION

An intervention package based on a situational analysis, consisting of a novel combined training and problem-solving approach and driven by HCWs, was able to significantly improve neonatal health care practices and reduce the injudicious use of many potentially harmful therapies. Future studies should target QI efforts at the full continuum of fetal-neonatal care including childbirth, resuscitation, transport, and postdischarge follow-up.

**Authors in the Retinopathy of Prematurity-Quality Improvement India Study Group**: All India Institute of Medical Sciences, New Delhi, India, Department of Pediatrics: Ashok K. Deorari and Anu Thukral; Postgraduate Institute of Medical Education and Research, Chandigarh, India, Department of Pediatrics: Praveen Kumar, Sourabh Dutta, Sundaram Venkataseshan, and Shiv Sajan Saini; Postgraduate Institute of Medical Education and Research, Chandigarh, India, Department of Ophthalmology: Mangat Dogra and Deeksha Katoch; Government Medical College and Hospital, Chandigarh, India, Department of Neonatology: Deepak Chawla, Suksham Jain, and Meenakshi Sharma; Government Medical College and Hospital, Chandigarh, India, Department of Ophthalmology: Subina Narang; Indian Institute of Public Health, Hyderabad, India: Rajan Shukla, G.V.S. Murthy, Snigdha Das, and Ammaji Ch; National Health Mission, Bhopal, India: Rajashree Bajaj and Manish Singh; and London School Hygiene & Tropical Medicine, London, United Kingdom, Department of Clinical Research: Clare Gilbert, International Centre for Eye Health.

## Notes

**Peer Reviewed**

**First published online:** October 11, 2022.
